# The ethics of artificial intelligence systems in healthcare and medicine: from a local to a global perspective, and back

**DOI:** 10.1007/s00424-024-02984-3

**Published:** 2024-07-06

**Authors:** Tijs Vandemeulebroucke

**Affiliations:** Bonn Sustainable AI Lab, Institut für Wissenschaft und Ethik, Universität Bonn—University of Bonn, Bonner Talweg 57, 53113 Bonn, Germany

**Keywords:** Artificial intelligence systems Healthcare Medicine, Bioethics, Global bioethics, Environmental ethics, Healthcare, Medicine

## Abstract

Artificial intelligence systems (ai-systems) (e.g. machine learning, generative artificial intelligence), in healthcare and medicine, have been received with hopes of better care quality, more efficiency, lower care costs, etc. Simultaneously, these systems have been met with reservations regarding their impacts on stakeholders’ privacy, on changing power dynamics, on systemic biases, etc. Fortunately, healthcare and medicine have been guided by a multitude of ethical principles, frameworks, or approaches, which also guide the use of ai-systems in healthcare and medicine, in one form or another. Nevertheless, in this article, I argue that most of these approaches are inspired by a local isolationist view on ai-systems, here exemplified by the principlist approach. Despite positive contributions to laying out the ethical landscape of ai-systems in healthcare and medicine, such ethics approaches are too focused on a specific local healthcare and medical setting, be it a particular care relationship, a particular care organisation, or a particular society or region. By doing so, they lose sight of the global impacts ai-systems have, especially environmental impacts and related social impacts, such as increased health risks. To meet this gap, this article presents a global approach to the ethics of ai-systems in healthcare and medicine which consists of five levels of ethical impacts and analysis: individual-relational, organisational, societal, global, and historical. As such, this global approach incorporates the local isolationist view by integrating it in a wider landscape of ethical consideration so to ensure ai-systems meet the needs of everyone everywhere.

## Introduction

As with every facet of society, healthcare and medicine are being confronted with the impacts of the rapid uptake of new forms of artificial intelligence, especially machine learning and generative artificial intelligence. Under the guise of promises of greater efficiency in different settings and practices, lowered healthcare costs, increased access, and better healthcare in general, artificial intelligence systems (ai-systems) are increasingly being developed for, implemented, and used in healthcare and medical settings and practices.

The introduction and increasing use of ai-systems are accompanied by various complex ethical tensions. Moreover, as healthcare and medicine explicitly deal with the health of people, and as such contribute to “the good life”, their practices are inherently ethically value-laden. Hence, the question becomes how the use of ai-systems will impact and mediate these practices and so also their inherent ethical dimension.

Fortunately, multiple established ethical principles testify to the fact that healthcare and medicine are inherently value-laden. Many of these principles have been integrated and operationalised in ethical frameworks that can guide the uptake of technological innovations, such as ai-systems. Nevertheless, most of these frameworks focus on the ethics of technological innovations once they are in existence and as such leave out multiple aspects of their full life cycles which are also in need of ethical scrutiny [59]. When it comes to ai-systems, which are usually conceived as purely digital technologies, we also see this tendency to only ethically evaluate these systems once they are in existence and we are forced to confront them. However, as previous research has shown, these systems are vulnerable to social biases, impinge on people’s privacy, lead to new power differentials in healthcare and medical contexts, and consume large amounts of resources and energy to name just a few ethical issues [65]. These issues need to be addressed not only once the systems are ready to be used, but also during their design, development, and other stages of their life cycles. Admittedly, approaches or frameworks to include some or all of these stages within ethical discourses on ai-systems in healthcare do exist [15], such as value sensitive design (VSD) [13, 60] and responsible research and innovation (RRI) [7, 46]. Nevertheless, it remains the question if these broader approaches to the ethics of ai-systems in healthcare and medicine meet the challenge of uncovering all related ethical issues and how all these issues relate to one another throughout the local and global dimensions of healthcare and medicine [59].

Hence, in this paper, I argue why it is urgent to broaden our perspective of the ethical landscape created by the introduction of ai-systems in healthcare and medicine. I will develop this broader perspective in three steps complemented by a concluding remark. In a first step, I provide a short overview of the current and predicted uses of these systems after which I develop two opposite characterisations of them. The first is a characterisation of a local isolationist nature, which emphasises the idea of ai-systems as isolated technologies which are used in local healthcare and medical systems and are detached from broader social and environmental contexts and considerations—a characterisation which I argue is currently very common in healthcare and medical settings. The second characterisation presents a picture of ai-systems as world objects going beyond the local isolationist perspective by including the multiple global networks entailing different environmental, technical, and social inputs which guarantee the possible existence of ai-systems.

Based on each of these characterisations, I present in a second step two approaches to the ethics of ai-systems in healthcare and medicine. The first is a local isolationist ethical approach, in this article exemplified in the commonly used principlist approach, which focusses on ethical issues occurring in a particular local setting, be it a particular care relationship, organisation, or society or region. I argue that despite this approach’s merits of enabling a practical means to resolve some ethical tensions induced by ai-systems in healthcare and medicine, it is insufficient once ai-systems are presented in their broader social and environmental contexts and the ethical issues that therein occur. Hence, I present a second global approach to the ethics of ai-systems in healthcare and medicine, which is a multi-level approach identifying five levels of ethical analysis: individual-relational, organisational, societal, global, and historical. This global approach incorporates the local isolationist approach and in doing so integrates ethical issues occurring within local health and medical settings with those occurring in social and environmental contexts in which these settings are located. Moreover, it interrelates the local and global dimensions of healthcare and medicine in general.

In a third and final step, I overview a few of the heavily debated ethical issues related to ai-systems in healthcare and medicine, organise them according to the presented global approach, and simultaneously indicate issues that currently receive much less attention. This leads me to conclude that ai-systems cannot, once their ethical tensions on a local level are resolved, simply be considered a positive contribution in healthcare and medical settings, but that they must also be considered, at least from an ethical perspective, as a potential new global health issue which is in urgent need of further analysis and critical reflection.

## Ai-systems in healthcare and medicine—a characterisation

### The use of ai-systems in healthcare and medicine

Automated systems in healthcare and medicine are not a recent phenomenon but can rather be traced back to the 1950s [42, 55] “[…] when physicians made the first attempts to improve their diagnoses using computer-aided programs” [55, p. 2]. Nowadays, when we speak of automation, ai-systems immediately come to mind. With the increasing availability of enormous amounts of digital data and the growing computational power of modern computers, we are experiencing an exponential upsurge of different ai-systems (e.g. machine learning systems, deep learning systems, natural language processing systems) in society in general and in healthcare and medicine in particular [55, 65].

This upsurge is accompanied by an explosion of research on concepts, developments, testing, and implementation of ai-systems in different healthcare and medical domains across the world [42, 48, 54, 55]. Among other settings, applications of ai-systems are being researched to be used in radiology, pathology, dermatology, ophthalmology, cardiology, emergency triage, and even mental health [16, 49, 53–55]. Despite this growing research interest, it seems that the clinical uptake and robust validation of these systems are difficult and currently lacking [16, 27, 34, 45, 53].

Nevertheless, there are high hopes that ai-systems will be beneficial for multiple healthcare and medical practices and settings. Following the World Health Organization (WHO) [65] and other authors [16, 42, 45, 53, 68], at least four of these practices and settings can be distinguished:

1. Diagnosis: It is well-agreed upon that ai-systems can positively impact diagnostics. Because of these systems’ capacity to analyse large amounts of data and find correlations between different data points, the use of these systems has the potential to reduce diagnosis time, catch diseases earlier, and better predict how diseases will develop over time, opening up a new area of preventive healthcare [53, 65, 68] and reduced workloads [16, 42]. Fields that rely on medical imaging and radiology, such as oncology, dermatology, and pathology, could especially benefit from such applications [42, 45, 53, 65, 68].
2. Clinical care: Analysing vast amounts of digital data makes ai-systems beneficial for clinical practices. The fact that different sources of patient information such as (electronic) health records and doctors’ notes could be integrated and streamlined increases the possibility to develop more robust treatment decisions, to avoid and catch clinical errors, and to improve clinical outcomes [42, 53, 65, 68]. Along this line of reasoning, it is also suggested that the use of ai-systems can lead to more personalised healthcare and medicine [45, 65, 68]. Moreover, ai-systems in tandem with other digital healthcare tools (e.g. healthcare apps) could lead to more remote healthcare formats [65, 68].
3. Healthcare administration: Ai-systems can be useful for processing healthcare and medical administration [42, 45, 53, 65, 68]. These systems can streamline administrative processes [68] and provide logistical support such as “[…] optimization of the medical supply chain, to assume mundane, repetitive tasks or to support complex decision-making […] identifying and eliminating fraud or waste, scheduling patients, predicting which patients are unlikely to attend a scheduled appointment and assisting in identification of staffing requirements” [65, p. 12]. These efficiency gains could lead to workload reductions and possibly to cost savings. Moreover, in the right circumstances, ai-systems could support the allocation of already restricted healthcare resources [45, 65].
4. Health monitoring: Ai-systems can also be applied to monitor individual and public health through the use of wearables and monitoring devices [65, 68]. These systems can be used to develop insight into causes of poor health or disease outbreaks such as environmental degradation [65] as well as the social and environmental determinants of health.

### A local isolationist view on ai-systems

With some insight into how ai-systems are being, and are predicted to be, used in healthcare and medicine, these systems can now be characterised. Among the plethora of different definitions of ai-systems in healthcare and medicine, at least two common traits can be discerned: (1) that these systems emulate (human) intelligent behaviour; and (2) that they are perceived from a locally isolated point of view.

The first trait, that ai-systems emulate (human) intelligent behaviour, is easy to spot. One just needs to look at how many policy documents and research publications, be they medically or ethically focused, refer to the fact that these systems appear to behave intelligently, and emphasise that they function with a certain level of autonomy [16, 20, 21, 27, 28, 42, 45, 54, 55, 65, 68]. For example, the WHO [65 p. 4], relying on a definition of the Organisation for Economic Co-operation and Development (OECD) [31], described an ai-system as “[…] a machine-based system that can, for a given set of human-defined objectives, make predictions, recommendations, or decisions influencing real or virtual environments. AI systems are designed to operate with varying levels of autonomy”. In their mapping review of the ethics of artificial intelligence in healthcare, Morley and colleagues [27 p. 1] refer to the “[…] classic definition of AI as an umbrella term for a range of techniques that can be used to make machines complete tasks in a way that would be considered intelligent *were* they to be completed by a human”. Meanwhile, Secinaro and colleagues [42, p. 1], in their review of ai-systems applications in healthcare, suggest that the concept “artificial intelligence” “[…] generally applies to computational technologies that emulate mechanisms assisted by human intelligence, such as thought, deep learning, adaptation, engagement, and sensory understanding” and that some “[…] devices can execute a role that typically involves human interpretations and decision-making”.

Besides being perceived as behaving intelligently, ai-systems are also perceived from a local and isolationist point of view. This second trait refers to the tendency to always conceive these systems from within a particular local setting and from a local perspective, be it the relationship between patient and healthcare professional, a particular healthcare organisation (e.g. hospital, nursing home), or a particular region or society. We speak about using ai-systems for our health and our healthcare and medicine, without defining who this “our” precisely is. The WHO shows awareness of this tendency when it makes a distinction between the different impacts ai-systems can have in low- and middle-income countries (LMIC) and high-income countries (HIC): ai-systems “[…] should be available for use not only in contexts and for needs in high-income settings but also in the contexts and for the capacity and diversity of LMIC” [65, p. xiii]. With this local perspective comes a perspective on ai-systems as “isolated entities” which often lacks “[…] a consideration of the wider contexts and the comprehensive relationship networks in which technical systems are embedded” [17, p. 103]. As I argue below, these relationship networks cover an individual, organisational, societal, global, and historical level. And because of this isolationist perspective, ai-systems appear to be (digital) objects that are imposed on us, that cannot be avoided, and which we now need to somehow adjust to and integrate in our local healthcare and medical settings.

### A global view on ai-systems

Recently, other characterisations of ai-systems have arisen which go beyond intellectual and local isolationist perspectives and emphasise these systems’ materiality. Under the pressure of growing evidence indicating different environmental costs, such as energy consumption [10, 33, 39], related CO_2_ and other greenhouse gasses emissions [24, 33, 39, 47], or water use [22, 23, 29, 33, 39] related to the development and use of ai-systems and the technical elements and material infrastructure needed for these systems to exist, increasing attention is being given to more holistic characterisations of ai-systems. For example, Green AI [41] presents an idea of ai-systems that explicitly takes computational environmental costs into account during the development phase and which proposes “efficiency” as a measure to get insight into these costs. Another example is van Wynsberghe’s [61, p. 217] idea of sustainable AI, aiming at fostering “[…] change in the entire lifecycle of AI products (i.e. idea generation, training, retuning, implementation, governance) towards greater ecological integrity and social justice”. Hence, sustainable AI indicates a conception of ai-systems beyond “[…] AI applications; rather, it addresses the whole sociotechnical system of AI”.

Here, I subscribe to van Wynsberghe and colleagues’ refinement of the idea of sustainable AI, by describing ai-systems as world objects [62]. With this description, they follow Feenberg’s [12] and Serres’ [43] conception of technologies as affecting the whole world, in its complete environmental and social understanding and not just one particular local place in it. By considering ai-systems as world objects, van Wynsberghe and colleagues [62] emphasise the relation between ai-systems and the necessary hardware and material infrastructure to run these systems. For them, ai-systems embody at least three interconnected global networks: (1) a global socio-environmental network referring to the necessary natural raw materials and labour force needed to produce and distribute the technical elements to guarantee the material infrastructure of ai-systems; (2) a global socio-material network covering the development of technical elements and the socio-technical relations that enable the development of ai-systems; and (3) a global digital network of software programs, computing analysis, ai-systems development, tuning, and use. As will become clear, conceived as world objects in this networked sense, ai-systems can be ethically analysed from each of the five levels—individual-relational, organisational, societal, global, and historical—to help uncover their environmental and social costs, which lead in turn to increased health risks for individuals and communities across the globe.

## From a local isolationist to a global ethics

Fortunately, the introduction and use of ai-systems in healthcare and medicine is not without its ethical guidance. Historically, healthcare and medical practices have been accompanied by ethical principles inspiring ideals of what good healthcare and medicine should be, what the role of a medical practitioner is, what the role of a patient is, etc. The Hippocratic Oath, for example, still plays its role in current healthcare and medical education and debates, albeit in different interpretations and versions [26].

Yet, the conception and development of the ethics of ai-systems in healthcare and medicine directly depends on how the systems themselves are conceived. In line with the distinction made between the local isolationist and the global view, I lay out a local isolationist and a global approach to the ethics of ai-systems in healthcare and medicine.

### A local isolationist ethics view on ai-systems

Nowadays, in a time characterised by ever-increasing technological innovations in healthcare and medicine, there are a multitude of ethical frameworks and traditions to assess and evaluate these systems [59]. However, despite this ethical diversity, it is clear that the biomedical principles of “respect for autonomy”, “beneficence”, “non*-*maleficence”, and “justice” covered by the umbrella notion of the “*principlist approach*” is still common today [59]. Together, these principles, popularised by the work of Beauchamp and Childress [4], focus on the particularity of doctor-patient relations and emphasise the individuality of each party in these relations.

Although it is undeniable that this approach has led to necessary ethical awareness in healthcare and medical settings and to a welcomed approach of how to practically deal with ethically loaded healthcare and medical situations (e.g. euthanasia, abortion, life-sustaining practices), it has created a number of blind spots. As ten Have [51] explains, the almost exclusive focus on the individual in the principlist approach to ethics in healthcare and medicine has created the idea that health and disease are an individual state of a person and therefore a purely individual responsibility. Additionally, it can be argued that the focus on the individual person has led to a local conception of healthcare and medicine that, although having strong societal implications, has been cut off from greater societal and global considerations. Nevertheless, “[…] social issues […] should not be overlooked […]” as healthcare and medicine are social practices “[…] affected by the social determinants of health (SDoH)” [8 , p. 2]. Healthcare and medicine also rely on different environmental determinants of health such as biodiversity which “[…] plays a critical role in ecosystem functioning and also yields direct and indirect benefits (or ecosystem services) that support human and social needs, including good health, food and nutrition security, energy provision, freshwater and medicines, livelihoods and spiritual fulfilment” [67, p. 26]. Hence, questions arise of how healthcare and medicine, along with the different practices and technologies they exist off, ethically relate to, for example, social issues such as poverty and inequality and to environmental crises such as biodiversity loss, climate change, and (e-)waste.

In the field of AI ethics, an abundance of ethical guidelines and principles have been utilised to guide the development, implementation, and use of ai-systems in different settings [19, 40]. Nevertheless, when it comes to ai-systems in healthcare and medicine in particular, the aforementioned principles of respect for autonomy, beneficence, non-maleficence, and justice still seem to play a dominant role in one form or another, regularly complemented by other principles such as explainability, explicability, fairness, transparency, responsibility, and/or trust [1, 2, 11, 15, 16, 20, 21, 25, 65]. However, here too, this principlist approach is related to a local individualised conception of healthcare and medicine, this time to a local isolationist perspective of ai-systems. Moreover, a principlist approach towards these technologies takes them for granted and as inevitable, and as such works from within the technical paradigm [6, 50, 57]. The best it can do is “[…] to establish ethical criteria which guarantee a careful design, development and use of AI, in order to avoid its sharp edges” [6, p. 6]. Hence, a principlist approach to the ethics of using ai-systems in healthcare and medicine is inspired by what Vandemeulebroucke and Bolte and their colleagues have called an “ethics of carefulness” and by a certain technological determinism [6, 57]. Moreover, because ai-systems are taken for granted and the role of ethics seems to have become merely the provision of ethical principles as assessment criteria, ethical issues related to these systems take the shape of technical issues which can be solved by technical means [17]. And similar to how the principlist approach to ethics in healthcare and medicine is insufficient to thoroughly deal with how healthcare and medicine ethically relate to social and environmental issues, this same approach to the ethics of ai-systems in healthcare and medicine is insufficient to deal with these issues.

### A global multi-level ethics view on ai-systems

This kind of principlist approach to the ethics of ai-systems in healthcare has its merits as it enables us to discover what these systems’ potential negative impacts on different healthcare practitioners and patients can be, so enabling design, development, and use that avoid these negative impacts as much as possible and increase the possibilities of their positive impact. However, because of its local and isolationist focus, this approach is less helpful once we conceive these technologies as world objects as described above.

In order to meet this world dimension of ai-systems, an ethical discourse needs to be developed that takes up a global perspective and that recognises that the individual patient, the individual care relation, and local healthcare and medical settings are contextualised in greater social and environmental structures. This leads to an ethical discourse that, in addition to clinical and medical ethics, also entails discourses such as environmental ethics, public health ethics, and social and economic ethics [58].

Fortunately, in the history of ethics of healthcare and medicine, such global perspectives have gained attention in discourses such as the ethics of environmentally responsible healthcare [32], green bioethics [36, 37], and global bioethics [35, 51, 52]. The latter, personified by Rensselaer Potter [35, p. 2], recognised in the late 1980s that with “[…] the focus on medical options, the fact that bioethics had been proposed to combine human values with ecological facts was forgotten by many: the ethics of how far to exercise technological options in the exploitation of the environment was not associated with the term bioethics”. I assert that this misrecognition still holds in most of the ethical debates taking place in healthcare and medicine, especially those regarding ai-systems. Hence, the “[…] time has come to recognize that we can no longer examine medical options without considering ecological science and the larger problems of society on a global scale” [35, p. 2]. As such, I agree with ten Have’s [51, p. 89] depiction of the task of global bioethics to “[…] improve the global context of health and the social structures within which care is provided” and that ethics “[…] is no longer the primary concern of individuals but also of communities, populations, states and transnational organizations”.

**Fig. 1:**
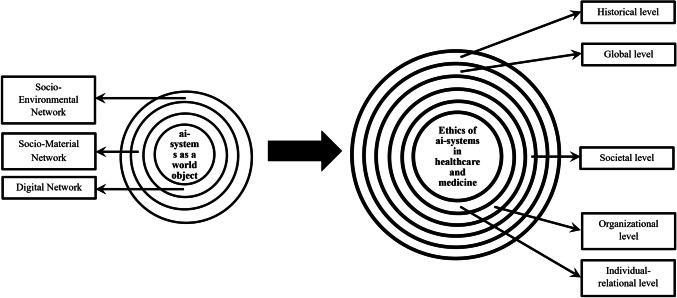
Conceptualisation of the global approach to the ethics of ai-systems in healthcare and medicine

Inspired by the global bioethics discourse as envisioned by Rensselaer Potter [35] and ten Have [51, 52], a foundation has been laid of what can be called a global approach to the ethics of ai-systems in healthcare and medicine [58]. To get insight into what this global approach practically entails, we can interpret it as embodying at least five interrelated levels of ethical analysis and impacts: the individual-relational, the organisational, the societal, the global [58], and the historical [56].^1^ Hence, a global approach to the ethics of ai-systems in healthcare and medicine encapsulates the local isolationist focus of the principlist approach and integrates it into broadening levels of ethical analysis and impact, ultimately linking different local and global health and ethics dimensions (Fig. 1).

## The ethics of ai-systems in healthcare and medicine—a broadened landscape

The landscape of the ethics of ai-systems in healthcare and medicine has been well-developed in recent years. Not only an abundance of relevant ethical principles has been identified, but also multiple potential ethical tensions instigated by the use of ai-systems [1, 11, 15, 16, 20, 21, 27, 28, 45, 65].

### The local isolationist landscape

Looking over this landscape, it seems that the majority of ethical analyses of the use of ai-systems in healthcare and medicine have been carried out from a local and isolationist perspective, mainly involving the individual-relational, organisational, and societal levels of ethical impacts and leaving the global and historical levels aside. Here, a few of these issues are presented in an indicative overview.

On an individual-relational level, we can for example consider how the ai-systems will impact patients’ and healthcare professionals’ privacy [1, 11, 15, 16, 20, 21, 28, 45, 65], autonomy [15, 16, 20, 27, 65], or dignity [11, 21, 45]. For ai-systems to enable personalised medicine or predictive diagnostics, they necessitate a continuous influx of patients’ data. Additionally, if these systems are meant to be used to optimise healthcare workloads and workflows, they will need to be trained by analysing these loads and flows so as to propose possible efficiency gains. Both uses of these systems seem to increase the risk of infringing on individuals’ privacy. Ai-systems can also impact patients’ and healthcare professionals’ autonomy as both could become less meaningfully involved in healthcare or medical decision-making [20, 27]. It is also not farfetched to imagine that healthcare professionals could feel violated in their professional dignity when these systems start to take over certain tasks [45], or at least transform their roles [11, 21, 27]. To deal with these and other issues, the described principles of respect for autonomy, beneficence, non-maleficence, and justice, complemented with principles such as explicability and transparency, are well suited as they enable us to describe these issues in a common language grounded in ethical principles and lead us to balanced positions between pros and contras.

Also viewed from an organisational level, ai-systems are meant to increase efficiency on the work floor and in the care provided. But here too, ethical issues arise. For example, the possibility exists that healthcare professionals will over-rely on the recommendations given by ai-systems because these systems are technologies and as such are presented and perceived as more reliable, more objective, etc., inducing what is known as automation bias [11, 16, 27, 28, 45]. The opposite, what can be called human bias, also occurs when healthcare professionals do not want to rely on these systems because they are technologies and as such are considered to be cold, quantitative, efficiency directed, etc. [28, 45]. Organisations need to reflect from their own perspective on good care, sometimes written out in mission statements and deontological codes, if ai-systems are usable, and if so, which functions these systems should fulfil and which they should not. Nevertheless, Sparrow and Hatherley [45, p. 97] rightly point out that failure “[…] to employ the best system will harm patients and so every institution will be under a moral obligation to adopt the best AI […]” which could instigate an endless competitive race between healthcare and medical organisations, but also between countries [28, 65], as new and better-equipped systems will come on the market in the foreseeable future [16, 45].

Finally, on a societal level, we can analyse the much-discussed issue of bias in the data used in training ai-systems and how these systems can perpetuate these biases [1, 11, 15, 16, 20, 21, 27, 28, 65]. As data comes forth out of social and historical contexts, the issue of data bias reveals unjustified historical social structures that also ground our healthcare, medical structures, and medical sciences. As the WHO [65, p. 55] states, many “[…] data sets used to train AI models are biased, as many exclude girls and women, ethnic minorities, elderly people, rural communities and disadvantaged groups”. Hence, their call to ensure inclusiveness and equity by imploring AI developers to “[…] be aware of the possible biases in their design, implementation and use and the potential harm that biases can cause to individuals and society” [65, p. 29]. Moreover, with the implementation of ai-systems in different healthcare structures, a new stakeholder is introduced, namely the providers of these systems. Consequently, the power relations between all healthcare and medical stakeholders are being and will continue to be reshaped [16, 27, 28, 45, 65]. Nevertheless, ai-systems “[…] should minimize inevitable power disparities between providers and patients or between companies that create and deploy AI technologies and those that use or rely on them”; everyone “[…] should be able to benefit from an AI technology and not just the technology providers” [65, p. 29–30].

### The global landscape

All of these aforementioned issues could and should also be analysed from a global perspective. Indeed, as indicated, competition to access the best ai-systems can occur between healthcare organisations, which can be analysed from an organisational level, but also between countries, which can be analysed from a global level. Bringing these issues to a global level leads us to reconsider how we interpret them. How do we conceive individual patients and healthcare professionals’ autonomy through this global lens? What does data bias in ai-systems entail from a global level?

Although these and other questions need critical analysis, here, I will focus on how the global level of the ethics of ai-systems in healthcare and medicine confronts us with questions of the ecological sustainable character of all healthcare and medical practices, and as such also of these systems. Indeed, like any sector in society, healthcare and medical settings also have a material and climate footprint, of which the burdens and the benefits are unequally distributed over different regions, societies, and communities across the world. For example, in regard to the climate footprint of healthcare and medicine, the WHO [64, p. 32] indicates that on “[…] the one hand, thousands of health centres across low- and middle-income countries are not connected to the grid and lack electricity, while, on the other hand, the global health care climate footprint makes up nearly 5% of greenhouse gas emissions”. This also holds for other resources such as mineral use to develop health technology, as well as the resulting e-waste, etc. The question then arises of how the use of ai-systems will impact the natural environment and the already existing global health inequities.

As evidenced by the small volume of existing research on the topic, the emission of greenhouse gasses, and thus the climate impact, of the development and use of ai-systems in healthcare and medicine in ethical analyses is just beginning to be explored [37, 44, 65]. Nevertheless, as shown, the development and the use of ai-systems and their technical elements and material infrastructure require high amounts of energy [10, 33, 39, 47] and contribute to the emissions of great amounts of greenhouse gasses [24, 33, 39, 47]. In a time characterised by a climate crisis [64] and an environmental crisis [67], it is mandatory to critically approach this consumption. As the WHO [65, p. 30] indicates, “[…] AI systems should be designed to minimize their ecological footprints and increase energy efficiency, so that use of AI is consistent with society’s efforts to reduce the impact of human beings on the earth’s environment, ecosystems and climate”. Moreover, climate change induces negative health outcomes such as “[…] death and illness from increasingly frequent extreme weather events, such as heatwaves, storms and floods, the disruption of food systems, increases in zoonoses and food, water, and vector-borne diseases, and mental health issues […]” and undermines “[…] many of the social determinants for good health, such as livelihoods, equality and access to healthcare and social support structures” [64, p.2].

Nevertheless, solely focusing on the amount of greenhouse gasses emitted by the development and use of ai-systems, and as such on their possible negative environmental and health impacts, would be a reductionistic perspective [37]. Indeed, as the conception of ai-systems as world objects indicates, other impacts of the material infrastructure required to develop and use these systems need to be included in our ethical assessments and evaluations. This infrastructure demands a steady supply of minerals and ores (e.g. gold, silver, copper) which are mined in different parts of the world. Mining practices have grave impacts on local natural environments and on the communities reliant on them. Mining practices lead to the loss of local nature and biodiversity by the practice itself, but for example also by the development of an infrastructure to transport the dug-up minerals, the pollution of local soils, water supplies, and the air by the use of toxic or other materials to free up the minerals, or by the improper closure of mines [5, 67]. Moreover, most of these practices are carried out in highly questionable labour conditions [5]. Similar environmental and social concerns exist with the management of the unavoidable e-waste that accompanies the digitalisation of societies and hence also by the contribution of ai-systems in healthcare and medicine. And these concerns will most likely only rise in the coming years as it is estimated that the amount of e-waste will grow to 74.7 million tonnes per year by 2030 (in comparison to the 53.6 million tonnes in 2019), of which currently only a small amount (17.4%) is managed in proper formal waste management systems (e.g. recycling) [14, 63]. The majority of this e-waste is informally processed by primitive practices “[…] which may include burning, heating or soaking in chemical baths” [63, p. 4] carried out by a labour force of which a significant part consists of women and children [63]. Lastly, as indicated before, the development and use of ai-systems require huge amounts of water [23]. Additionally, the development of the necessary technical elements (e.g. semi-conductors) and the material infrastructure to develop ai-systems require enormous amounts of water [22, 29, 33, 39]. For example, in 2019, one of the biggest developers and suppliers of semi-conductors in the world consumed up to 63 million tons of water across its regional facilities and so, despite its water recycling practices, increased the risk of local droughts [3, 69].

All of these environmental and social impacts of ai-systems affect most heavily those local communities that are least likely to reap all the possible benefits of digitalisation [5, 63], such as the use of ai-systems in healthcare and medicine [66], because, for example, of a lack of the necessary material infrastructure (e.g. robust electricity grid). These communities exist on a global level, but also within particular nations and regions. Moreover, each of these and other environmental and social impacts come with increased health risks, quite similar to the increased risks induced by climate change, such as increased risks of death, a possible increase of vector-borne diseases, a higher probability of cancers, and lung damage [5, 30, 63, 67]. In the end, these environmental and social impacts, and the increased health risks that accompany them, lead us to question whose health we are and should be considering when we are discussing the use of ai-systems in healthcare and medicine. Are we solely considering the patient in front of us, or do we also include the health of those people around the world who are affected by the development of the ai-systems we use in our local healthcare settings?

Finally, these environmental and social considerations are also relevant when considering how the use of ai-systems in healthcare and medicine will impact those people who will come after us. This historical level of ethical analysis and impact leads us to questions such as “What kind of healthcare and medicine do we want to leave behind for future generations? What world in which this healthcare and medicine takes place do we want to leave behind?” To deal with these questions, the principle of intergenerational justice can guide us [18]. While it is a difficult task to figure out what we owe to future generations, it is good to be aware of the fact that each choice we make in the present will in some way or another impact those people not yet alive. At least based on the reflections presented here, precaution is warranted. Moreover, these questions and reflections require an answer *now*, while the integration of ai-systems, at least the new generation of these systems, is still underway and changeable, so to avoid “[…] blindly going forward with the creation of a dependence relationship on a technology whose environmental impact, based on the little we do know, is extremely high” [38, p. 11].^2^

## Conclusion

Ai-systems have been a part of particular healthcare and medical settings for a long time. With recent technical developments, the technology has received exponential attention yet once again. In this article, a brief overview was given of the current and potential use of these systems. In short, it seems that the possibilities are nearly endless. Indeed, the promises and expectations are high: ai-systems are expected to make healthcare and medicine more clinically accurate, more efficient, more cost-effective, and hence more accessible, etc.

However, the use of ai-systems in healthcare and medicine is not without ethical concern. This concern, I argue, is not so much because of people’s fear of the automating aspect of ai-systems, but rather because of the inherent historical value-ladenness of healthcare and medicine and their practices. This value-ladenness has been embodied in different codes, frameworks, and ethical traditions and approaches enabling the identification and resolution of different ethical issues related to the use of ai-systems. Although necessary and laudable, it does appear that most current approaches to the ethics of ai-systems in healthcare and medicine are characterised by a local isolationist focus, both on ai-systems and the healthcare and medical settings in which these are to be used, which results in the obfuscation of broader societal and environmental structures.

Hence, this article proposed a global approach to the ethics of ai-systems that integrates this local isolationist focus into ever-expanding levels: individual-relational, organisational, societal, global, and historical. By giving an indicative overview of already identified ethical issues related to ai-systems according to these levels and by complementing them with less well-known and discussed issues, this article has sought to provide insight into the use of ai-systems in healthcare and medicine as a practice with simultaneously positive and negative, and local and global, ethical and health impacts.

Only by gaining insight into this dynamic between the local and the global are we confronted with our own responsibility to this dynamic and thus can the development and use of ai-systems be as ethically responsive as possible. Moreover, the ongoing digitalisation yet again confronts us with the question of how healthcare and medicine relate to the different social and environmental contexts and how sustainable healthcare and medicine can be guaranteed for all humans, whether they are close to us, the local; far away from us, the global; or yet to come into existence, the historical.

## Data Availability

No datasets were generated or analysed during the current study.

## Footnotes

1 Other multi-level approaches to the ethics of ai-systems in healthcare and medicine can be found in Morley and colleagues [27] and Smallmann [44].

2 This future looking ethical analysis should also be complemented by a historical-ethical analysis. For such a historical-ethical analysis in relation to the use of care robots in healthcare, see Coeckelbergh [9].
